# Long Non-Coding RNA GAS5 and Intestinal MMP2 and MMP9 Expression: A Translational Study in Pediatric Patients with IBD

**DOI:** 10.3390/ijms20215280

**Published:** 2019-10-24

**Authors:** Marianna Lucafò, Letizia Pugnetti, Matteo Bramuzzo, Debora Curci, Alessia Di Silvestre, Annalisa Marcuzzi, Alberta Bergamo, Stefano Martelossi, Vincenzo Villanacci, Anna Bozzola, Moris Cadei, Sara De Iudicibus, Giuliana Decorti, Gabriele Stocco

**Affiliations:** 1 Institute for Maternal and Child Health—IRCCS “Burlo Garofolo”, 34137 Trieste, Italy; marianna.lucafo@burlo.trieste.it (M.L.); matteo.bramuzzo@burlo.trieste.it (M.B.); annalisa.marcuzzi@burlo.trieste.it (A.M.); sadeiu@libero.it (S.D.I.); 2 PhD School in Science of Reproduction and Development, University of Trieste, 34127 Trieste, Italy; letizia.pugnetti@libero.it (L.P.); dcurci@units.it (D.C.); alessia.disilvestre@gmail.com (A.D.S.); 3 Callerio Foundation Onlus, 34127 Trieste, Italy; a.bergamo@callerio.org; 4 Cà Foncello Hospital, 31100 Treviso, Italy; stefano.martelossi@aulss2.veneto.it; 5 Pathology Section, Spedali Civili, 25123 Brescia, Italy; vincenzo.villanacci@asst-spedalicivili.it (V.V.); anna.bozzola@gmail.com (A.B.); moriscadei@vodafone.it (M.C.); 6 Department of Medicine, Surgery and Health Sciences, University of Trieste, 34127 Trieste, Italy; 7 Department of Life Sciences, University of Trieste, 34127 Trieste, Italy; stoccog@units.it

**Keywords:** inflammatory bowel disease, long non-coding-RNA, *GAS5*, *MMP2*, *MMP9*

## Abstract

Background: The long non-coding RNA (lncRNA) growth arrest–specific transcript 5 (*GAS5*) seems to be involved in the regulation of mediators of tissue injury, in particular matrix metalloproteinases (MMPs), implicated in the pathogenesis of inflammatory bowel disease (IBD). We investigated the role of *GAS5* in regulating *MMP2* and *MMP9* expression in pediatric patients with IBD and in vitro. Methods: In total, 25 IBD patients were enrolled: For each patient paired inflamed and non-inflamed biopsies were collected. RNA was extracted and *GAS5*, *MMP2*, and *MMP9* were quantified by TaqMan assay. The expression of *GAS5* and MMPs was also determined in the human monocytic THP1 cells differentiated into macrophages and stimulated with lipopolysaccharide (LPS). The function of *GAS5* was assessed by overexpressing the lncRNA and evaluating the MMPs levels. Results: Real-time PCR results demonstrated a downregulation of *GAS5* and an upregulation of both MMPs in inflamed tissues. In vitro data confirmed the trend observed in patients for the three genes: The stimulation with LPS promoted a downregulation of *GAS5* while an increase of MMPs was observed. Overexpression experiments showed that higher levels of *GAS5* lead to a decrease of both enzymes. Conclusion: These results provide new information about the role of *GAS5* in IBD: The lncRNA could mediate tissue damage by modulating the expression of MMPs.

## 1. Introduction

Inflammatory bowel disease (IBD) includes two different pathologies, Crohn’s disease (CD) and ulcerative colitis (UC), that, although different in their pathogenesis, show common clinical characteristics such as chronic inflammation at different levels of the gastrointestinal tract and a dysregulated excessive immune response [1]. Both CD and UC are characterized by chronic changes in the intestinal tissues, consisting of an intense infiltration of lymphocytes and plasma cells and deep remodeling of the connective tissue, leading to increased turnover of extracellular matrix (ECM) components [2]. Disturbance of the balance between the synthesis and degradation of ECM elements may result in progressive intestinal barrier destruction.

Degradation of ECM components is tightly controlled by the enzymatic activity of matrix metalloproteinases (MMPs), proteins that play an important role in the pathogenesis of IBD [3]. MMPs are secreted as pro-enzymes and require cleavage of the pro-peptide to become active. Increased levels of these proteins in the intestinal mucosa of IBD patients have been reported: The epithelial-derived *MMP9* is an important mediator of tissue injury in colitis, whereas *MMP2* maintains gut barrier function, favoring infiltration processes of leukocytes in inflamed tissue [4,5,6,7,8].

Long non-coding RNAs (lncRNAs) have emerged as important gene expression regulatory elements and recent data have implicated the deregulated expression of certain lncRNA networks in the pathogenesis of autoimmune and inflammatory diseases, such as IBD [9,10,11]. In this context, one of the most studied lncRNAs is growth arrest–specific transcript 5 (*GAS5*). *GAS5* can be deregulated by different pro-inflammatory stimuli in vitro [12,13,14]. Moreover, *GAS5* attenuates the expression and the activity of *MMP2* and *MMP9* and has a role in regulating the metastasis phenotype of melanoma cells [15,16]; however, no data are published about the role of *GAS5* as a regulator of *MMP9* and *MMP2* expression in IBD patients.

In the present study, our research focused on the potential role of *GAS5* in regulating the transcription of *MMP2* and *MMP9*, starting from their evaluation in inflamed and non-inflamed colonic tissues of pediatric patients with IBD. Moreover, considering that activated monocytes and macrophages are the major contributors of *MMP9* and *MMP2* in inflammatory diseases [17,18], an in vitro study on these cellular models was done to investigate the association between *GAS5* and the two MMPs’ expression.

## 2. Results

### 2.1. Patients

Twenty-five children with IBD (CD 13; UC 12) were enrolled at diagnosis in this study and their baseline characteristics and histologic findings are shown in Table 1.

### 2.2. GAS5 Expression in Mucosal Biopsies of Pediatric IBD Patients

**Figure 1:**
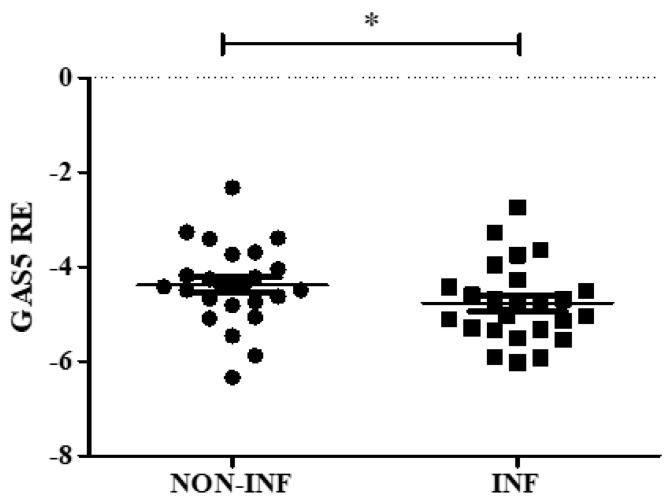
Growth arrest–specific transcript 5 (*GAS5*) levels in colon biopsies of inflammatory bowel disease (IBD) pediatric patients. Expression was evaluated in inflamed (INF) and non-inflamed (NON-INF) tissues for each patient. *GAS5* expression was normalized using *RPLP0* gene. Relative expression (RE) values are expressed as Log_2_ 2^−ΔCt^. Paired *t* test * *p* < 0.05.

For each patient, during colonoscopy, an inflamed and a non-inflamed biopsy was collected in TRIzol reagent. *GAS5* gene relative expression was evaluated, showing a statistically significant downregulation in inflamed mucosa in comparison to the non-inflamed one (Figure 1 and supplementary Table S1).

#### *GAS5* Correlation with Disease Activity Scores

**Figure 2:**
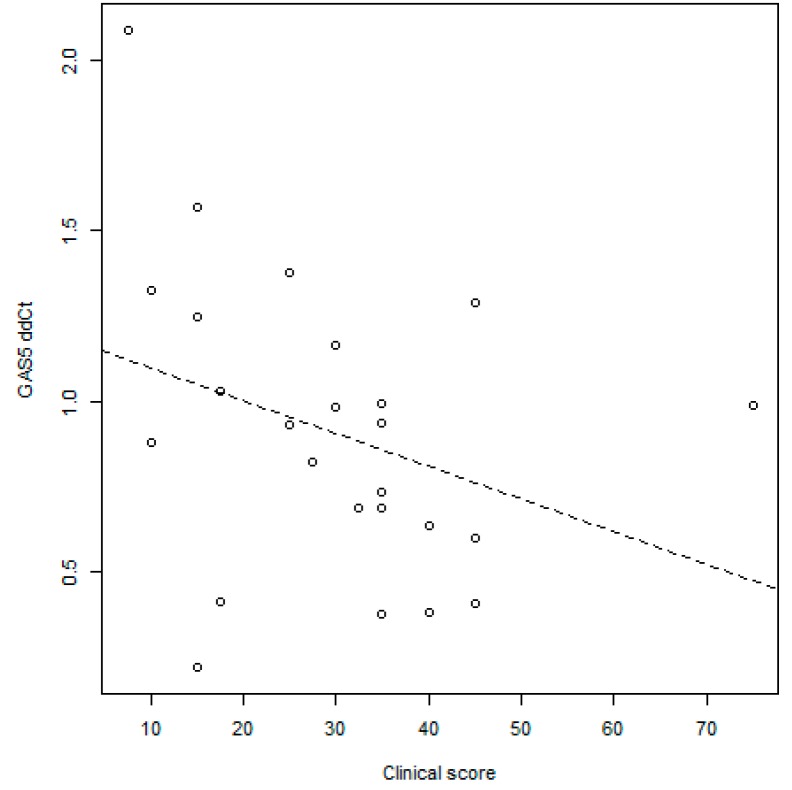
Correlation between *GAS5* levels evaluated in inflamed tissue and the clinical score; Spearman test *p* = 0.048; *r* = −0.40.

Interestingly, *GAS5* levels were negatively associated with the clinical scores reported in Table 1. No significant association was found with the inflammation and architectural parameters (Figure 2).

### 2.3. MMP2 and MMP9 Gene and Protein Expression in Colon Biopsies of Pediatric IBD Patients

**Figure 3:**
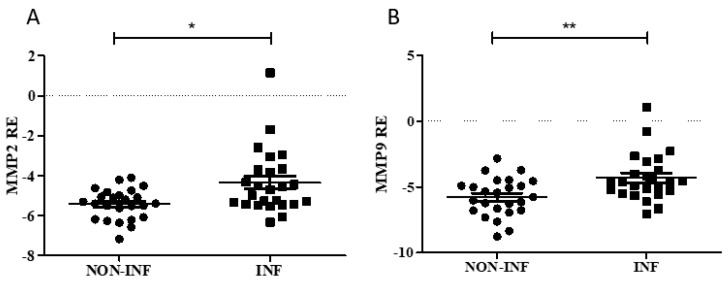
MMP (matrix metalloproteinase) levels in colon biopsies of IBD pediatric patients. *MMP2* (**A**) and *MMP9* (**B**) expression was evaluated in inflamed (INF) and non-inflamed (NON-INF) tissues for each patient and normalized using *RPLP0* gene. Relative expression (RE) values are expressed as Log_2_ 2^−ΔCt^. Paired *t* test * *p* < 0.05, ** *p* < 0.01.

Gene and protein expression analyses were performed to study the relation between the enzymes and *GAS5* in colon biopsies of pediatric patients with IBD at diagnosis. Real-time PCR results demonstrated an increased expression of both MMPs in inflamed tissues (Figure 3 and supplementary Table S1).

**Figure 4:**
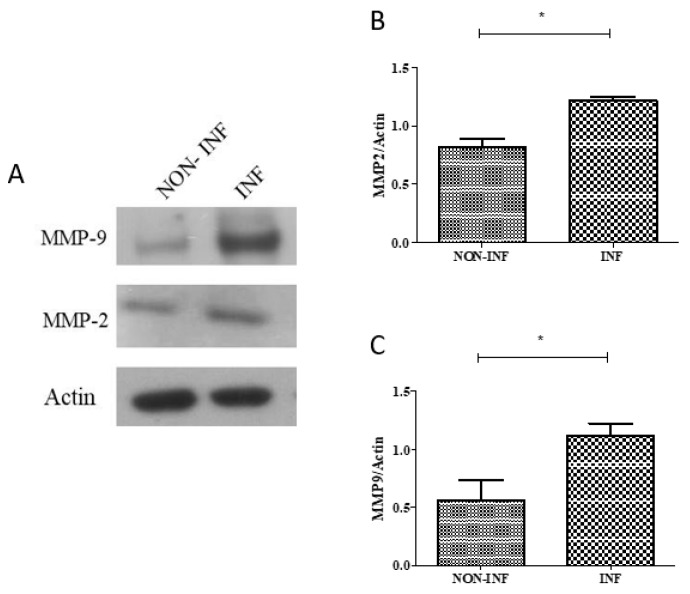
*MMP2* (74 kDa; **A** and **B**) and MMP9 (92 kDa; **A** and **C**) protein expression in colon biopsies of IBD pediatric patients analyzed by immunoblotting. Protein lysates were obtained from inflamed (INF) and non-inflamed (NON-INF) tissues. Actin (42 kDa) was used as internal loading control. Representative western blot reflecting *MMP2*, *MMP9* and actin protein levels. Paired *t* test * *p* < 0.05.

Immunoblotting analysis of the *MMP9* and *MMP2* proteins was also performed on inflamed and non-inflamed biopsies of three additional patients (mean age at enrolment 13.8 years, two UC and one CD, two males and one female). An increase in the expression of both proteins in inflamed tissues compared to non-inflamed ones was observed (Figure 4).

### 2.4. GAS5, MMP2, and MMP9 Gene Expression in the THP1 Cell Line

**Figure 5:**
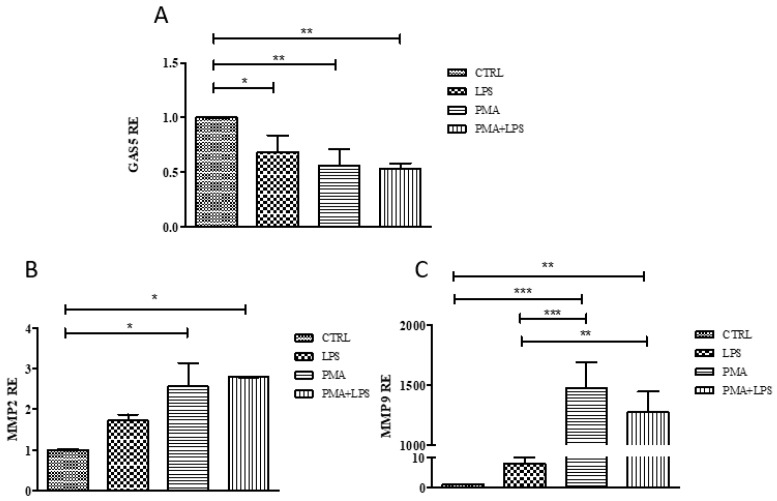
Relative expression (RE, values are expressed as 2^−ΔCt^) of *GAS5* (**A**), *MMP2* (**B**), and *MMP9* (**C**) in THP1 cells exposed to LPS (lipopolysaccharide), PMA (phorbol-12-myristate 13-acetate), and PMA + LPS. *GAS5*, *MMP2*, and *MMP9* expression was normalized using *18S* gene. One-way ANOVA *GAS5 p* = 0.003, *MMP2 p* = 0.018, *MMP9 p* < 0.0001 Bonferroni’s multiple comparison post-test * *p* < 0.05, ** *p* < 0.01, *** *p* < 0.001.

The THP1 human monocytic cell line was chosen as the cellular model to confirm the involvement of *GAS5* in the regulation of *MMP2* and *MMP9* expression. Experiments were conducted at different stages of differentiation, from monocyte (THP1) to macrophages (THP1 + PMA (phorbol-12-myristate 13-acetate)). The effects of stimulation by LPS was also tested. Real-time PCR results of GAS5 expression showed a downregulation of its levels in stimulated cells compared to unstimulated THP1. A decrease of *GAS5* was also evident in PMA-differentiated macrophages compared to monocytes, while LPS treatment did not further reduce *GAS5* expression (Figure 5).

*MMP2* and *MMP9* expression levels increased in stimulated cells, particularly in PMA-differentiated macrophages, with a similar trend for both MMPs (Figure 5), confirming the trend observed in patients.

### 2.5. MMP2 and MMP9 Gene Expression in Cells Transfected with the pcDNA3.1-GAS5 Plasmid

**Figure 6:**
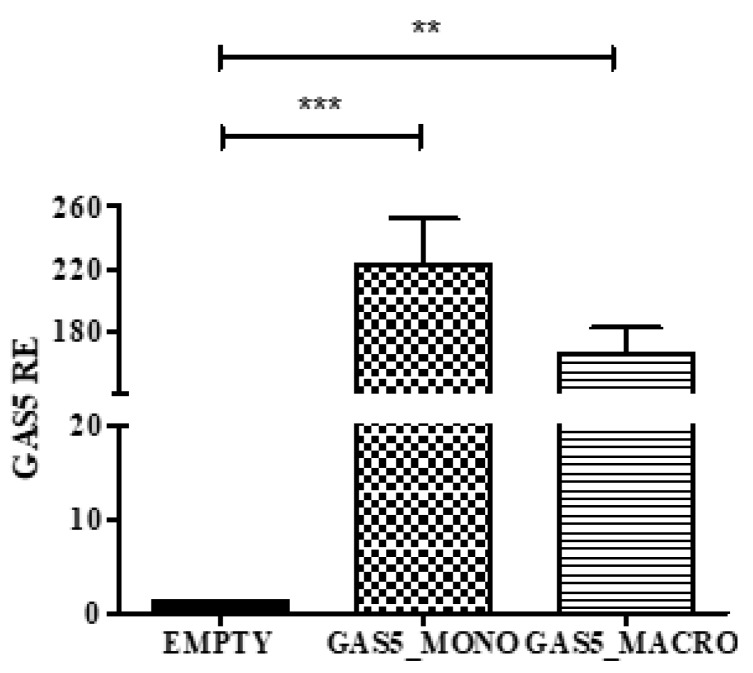
Evaluation of *GAS5* levels after transfection with pcDNA-*GAS5* plasmid in monocytes (*GAS5*_MONO) and macrophages (*GAS5*_MACRO). GAS5 expression was normalized using *GAPDH* gene and relative expression (RE) values are expressed as 2^−ΔCt^. One-way ANOVA *GAS5 p* = 0.0005, Bonferroni’s multiple comparison post-test ** *p* < 0.01, *** *p* < 0.001.

Following the transient transfection with pcDNA-*GAS5* plasmid, the efficiency of the overexpression of the lncRNA on both monocyte (THP1) and macrophages (THP1 + PMA) was assessed by real-time PCR (Figure 6).

**Figure 7:**
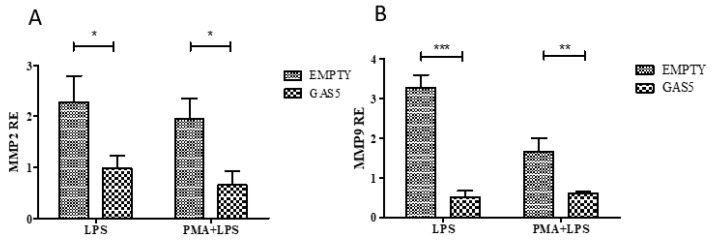
Levels of MMP2 (**A**) and MMP9 (**B**) after overexpression of GAS5 (GAS5) in monocyte stimulated with LPS (LPS) and macrophages stimulated with LPS (PMA + LPS) and in controls (EMPTY). MMP2 and MMP9 expression were normalized using *GAPDH* gene and relative expression (RE) values are expressed as 2^−ΔΔCt^. Two-way ANOVA: EMPTY vs. GAS5 MMP2 *p* = 0.002, MMP9 *p* < 0.0001 Bonferroni’s multiple comparison post-test * *p* < 0.05, ** *p* < 0.01, *** *p* < 0.001.

Real-time PCR results of *MMP2* and *MMP9* expression, reported in Figure 7, showed a downregulation in LPS-stimulated monocytes and macrophages compared with the respective control transfected with the empty vector, demonstrating the involvement of the lncRNA in the regulation of gene expression of the two MMPs.

### 2.6. MMP2 and MMP9 Proteins Released by THP1 Cells Overexpressing GAS5

**Figure 8:**
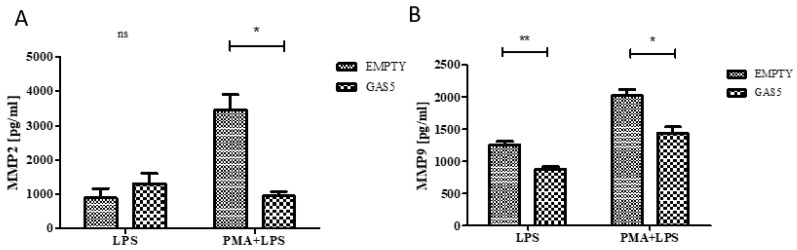
Evaluation of levels of MMP2 (A) and MMP9 (B) after overexpression of GAS5 (GAS5) in monocyte stimulated with LPS (LPS) and macrophages stimulated with LPS (PMA + LPS) and control (EMPTY). Two-way ANOVA: EMPTY vs. GAS5 MMP2 *p* = 0.02, MMP9 *p* < 0.0001 Bonferroni’s multiple comparison post-test * *p* < 0.05, ** *p* < 0.01; ns = not significant.

To confirm the role of *GAS5* in regulating the expression of *MMP2* and *MMP9*, their levels in the supernatant of both LPS-stimulated monocytes and PMA-differentiated macrophages overexpressing *GAS5* were evaluated by enzyme-linked immunosorbent assay (ELISA) test. As reported in Figure 8, *MMP9* was less expressed in *GAS5*-transfected monocytes with respect to the control; no difference was detected measuring MMP2 levels. Overexpressing *GAS5* significantly downregulated both MMP proteins in macrophages stimulated with LPS compared to the cells transfected with the empty vector (Figure 8).

## 3. Discussion

In IBD pediatric patients, genetic and environmental factors are responsible for the alteration in the epithelial barrier; the activity of pro-inflammatory elements released from macrophages, T cells, and innate lymphoid cells is also important [19]. MMPs have a key role in this context: In addition to playing a central role in ECM turnover, they activate or degrade a variety of other substrates including chemokines, cytokines, growth factors, and junctional proteins [20].

Several studies have shown that *MMP2* and *MMP9* are highly expressed in IBD inflamed colonic mucosa, serum, urine, and fecal samples [7,21,22,23]. However, the molecular mechanism by which the levels of these enzymes can be modulated during the inflammatory response still remains unclear.

Recently, it has been suggested that lncRNAs may play an important role in the pathophysiology of IBD, and many researches have therefore covered this topic. Forty-seven lncRNAs dysregulated in IBD were identified, and, even though their exact role requires more studies, it has been suggested that they might have a crucial role in the regulation of inflammatory pathways [24].

The present study explores the role of the lncRNA GAS5 as a regulator of two important mediators of inflammatory response in IBD: MMP2 and the MMP9. In particular, we evaluated the expression of *GAS5*, *MMP2*, and *MMP9* in biopsies of drug-naïve patients with CD and UC. Our results showed that the expression of the lncRNA in patients with IBD was lower in inflamed tissues compared to the adjacent normal part, while the expression levels of *MMP2* and *MMP9* increased in inflamed biopsies, confirming previously published results [3,4,5,22,23].

As reported in the literature, GAS5 seems to play a role in various inflammatory and autoimmune diseases such as rheumatoid arthritis and systemic lupus erythematosus in which significantly reduced *GAS5* levels in immune cells were shown [25,26]. Moreover, the lncRNA is differently expressed in pediatric patients with IBD treated with glucocorticoids and positively correlates with steroid resistance, suggesting a role of *GAS5* in the modulation of drug efficacy [27].

Preliminary data have already shown that *GAS5* is involved in the regulation of these two MMPs, indeed Chen et al. have shown that the expression of MMP2 and MMP9 is inversely correlated with the levels of the lncRNA *GAS5* in melanoma cells [15]. Overexpression of *GAS5* reduced the levels of the proteins whereas the knockdown increased their expression [15].

Our work is the first report in which the potential role of *GAS5* in the regulation of MMPs in IBD patients is described. Interestingly, data obtained from our cohort highlight a negative correlation between *GAS5* levels and the clinical scores PUCAI (Pediatric Crohn’s Disease Activity Index) and PCDAI (Pediatric Ulcerative Colitis Activity Index). These scores currently are the most rigorously developed non-invasive disease activity indexes in the field of childhood IBD, combining subjective reporting of the degree of abdominal pain, stool pattern, and general well-being and laboratory test results (hematocrit, erythrocyte sedimentation rate, and serum albumin) into a single score. PUCAI and PCDAI scores are significantly higher in patients with active disease while a score of <10 is consistent with inactive disease [28]. Endoscopy and clinical indexes are not always perfectly correlated because some symptoms are not related to the mucosal inflammation: *GAS5* levels could be a specific molecular marker useful to better predict disease activity and stratify the severity of the disease. This result further highlights the need to investigate the potential function of *GAS5* in IBD pathogenesis and progression. Currently, significant correlations between two lncRNAs quantified in the ileum of CD patients, LINC01272 and HNF4A-AS1, and more severe mucosal injury were found, but no correlation with clinical disease activities has been reported [29]. Differently, for *GAS5*, no correlation was found with endoscopic and inflammation scores in our cohort of patients; however, the clinical score was also not associated with these parameters in our study, as already described [30].

Considering that remodeling of the ECM and cell surface by MMPs is one of the most important function of monocytes and macrophages we decided to investigate the role of *GAS5* in the regulation of *MMP* expression in the THP1 monocyte cell line. In particular, in vitro experiments at different stages of differentiation, from monocyte to macrophages, stimulated with a proinflammatory factor were conducted. The results confirmed the trend observed in patients for the three genes: Stimulation with LPS promotes a downregulation of *GAS5* in both monocytes and macrophages, while an increase of *MMPs* was observed. This data highlight that, in response to immune activation, *GAS5* is transcriptionally repressed but the mechanism involved needs to be further investigated.

We thus studied the effects of *GAS5*-overexpression on the production of MMPs in response to LPS stimulation in terms of gene and protein expression. Forced expression of *GAS5* in monocytes and macrophages stimulated with LPS led to a reduction of levels of MMPs in both RNA and proteins, supporting the role of *GAS5* in modulating their expression, probably at the transcriptional level. It would be interesting to evaluate in our cellular model whether the reduced expression of *GAS5* could induce an increase of *MMP2* and *MMP9* expression as already reported in other cells [15].

It is well known that lncRNAs can regulate gene expression at the epigenetic, transcription, RNA processing, and translation levels [31]. The presence of *GAS5* both in the cytoplasm and the nucleus was confirmed in our previous study [27]. Functional studies have demonstrated that *GAS5* suppresses transcription by recruiting the histone methyltransferase polycomb repressive complex 2 to the promoter region of target genes, inhibiting gene expression [32]; further interaction studies by means of chromatin immunoprecipitation should be focused on this pathway to demonstrate the molecular mechanism by which *GAS5* regulates MMPs transcription.

Moreover, *GAS5* seems to have a protective role during the induction of inflammation: Its upregulation repressed the MMPs, acting as an anti-inflammatory agent. Actually, only one paper describing *GAS5* expression after LPS-induced inflammation was published. The authors found that *GAS5* was downregulated in ATDC5 chondrocyte cells and its overexpression alleviated LPS-induced injury [33]. Moreover, the expression levels of tumor necrosis factor (TNF)-α, interleukin (IL)-1β, IL-6, and IL-8 were all significantly lowered by *GAS5* overexpression. The mechanism proposed by the authors is that *GAS5* could modulate LPS-induced inflammatory damage through regulation of Krüppel-like factor 2 expression and inhibition of the nuclear factor kappa-light-chain-enhancer of activated B cells (NF-κB) pathway [33].

In conclusion, the present study provides new information about the functional role of *GAS5* in IBD patients: The lncRNA seems to be regulated by pro-inflammatory factors and could have an important role in mediating tissue damage by modulating the expression of *MMP2* and *MMP9*. In the future, the functional role of intestinal epithelium should be also investigated in this context using an innovative platform such as intestinal organoids.

Moreover, this study provides new perspectives to stratify IBD phenotype by using as a single measurement *GAS5* expression in biopsies and supplies novel targets for future drug development in IBD.

## 4. Materials and Methods

### 4.1. Clinical Samples and Histologic Evaluation

Twenty-five IBD pediatric patients were enrolled at diagnosis at the gastroenterology department of the Pediatric Clinic of IRCCS Burlo Garofolo in Trieste. For each patient, during colonoscopy, two biopsies of the endoscopically inflamed and non-inflamed areas were collected. TRIzol^®^ reagent was used for RNA isolation. Protein analysis was also performed on inflamed and non-inflamed biopsies obtained from three additional patients and immediately frozen on dry ice.

Histologic evaluation was performed for all patients enrolled in the study in which an adequate sampling was obtained according to European Crohn’s and Colitis Organisation (ECCO) guidelines (two biopsies for the five segments of the colon and terminal ileum) [34]. Histologic activity was described using a predefined histologic inflammatory score and an architectural abnormalities score, developed for a previous study [35].

Both inflammatory and architectural score were described with a range from 0 (absence of inflammatory activity) to 5 (maximal inflammatory activity) calculated considering three defined morphological elements:

- active inflammation (neutrophils aggressive toward the glandular structures in the lamina propria) (range from 0 to 3);
- crypt abscesses (range from 0 to 1);
- erosions and ulcerations (i.e., presence of granulation tissue) (range from 0 to 1).

Architectural abnormalities comprised three defined characteristics:

- glandular crypts alteration (range from 0 to 3);
- basal plasmacytosis (range from 0 to 1);
- epithelioid granulomas (range from 0 to 1).

### 4.2. Ethical Considerations

Local ethical committee approval for the study (Protocol number: 2198; 17th September 2013) was provided. The study was conducted in accordance with the principles outlined in the Declaration of Helsinki, and the parents of all the participating children gave written informed consent before the study began. Inclusion criteria were pediatric patients (5–18 years) with a diagnosis of CD or UC. Exclusions criteria were: a) patients already treated with immunosuppressive drugs at the diagnosis; b) disease requiring immediate surgical intervention; c) severe ulcerative colitis or toxic megacolon; d) any of the following conditions: active infection; stool culture positive for enteric pathogens; tumors; human immunodeficiency virus; transplanted organ; or non-controlled disease of the kidney, liver, endocrine system, heart, blood, nervous system, or brain.

### 4.3. Total RNA Isolation

Total RNA was extracted from biopsies and cells using TRIzol reagent (Thermo Scientific, Carlsbad, CA, USA) according to manufacturer’s instructions. The RNA concentration and purity were calculated by Nano Drop instrument (NanoDrop 2000, EuroClone, Milan, Italy).

### 4.4. Quantitative Real-Time PCR

Expression levels of *GAS5*, *MMP2*, and *MMP9* genes were evaluated by real-time RT-PCR TaqMan^®^ analysis using the CFX96 real-time system-C1000 Thermal Cycler (Bio-Rad Laboratories, Hercules, CA, USA). The reverse transcription reaction was carried out with the High Capacity RNA-to-cDNA Kit (Applied Biosystem, Foster City, CA, USA) and the real-time PCR was performed in triplicate using the TaqMan^®^ Gene Expression Assay to assess *GAS5*, *MMP2*, and *MMP9* mRNA expressions, according to the manufacturer’s instructions. The thermal cycling conditions for TaqMan assays were as follows: 2 min at 50 °C and 10 min at 95 °C followed by 40 cycles at 95 °C for 15 s and 60 °C for 60 s.

The expression levels of *GAS5*, *MMP2*, and *MMP9* were evaluated using the comparative Ct method (2^−ΔΔCt^ method). The *GAS5*, *MMP2*, and *MMP9* expression values were normalized using the *RPLP0* gene for studies encompassing tissues sampled from IBD patients [36] and the *18S* or *GAPDH* genes for the in vitro studies.

### 4.5. Immunoblotting

Inflamed and non-inflamed biopsies, obtained from the colon of IBD patients, were frozen, homogenized and then lysed using a lysis buffer composed of Tris-HCl 10 mM pH 7.4, EDTA 100 mM, NaCl 100 mM, SDS 0.1%, and protease inhibitor cocktail 1% (Sigma, Saint Louis, MO, USA). The protein concentration was determined using the Bradford reagent (Sigma, Saint Louis, MO, USA). Samples were run in a denaturing 10% polyacrylamide gel (Thermo Fisher Scientific, Carlsbad, CA, USA) and were transferred to a PVDF membrane (Thermo Fisher Scientific, Carlsbad, CA, USA) that was incubated overnight at 4 °C with primary antibodies (anti-actin 1:10000 (42 kDa), anti-MMP9 1:1000 (92 kDa), and anti-MMP2 1:1000 (74 kDa); Sigma Saint Louis, MO, USA). The secondary antibodies were an anti-mouse horseradish peroxidase (HRP) conjugated secondary antibody (Cell Signaling, Danvers, MA, USA), diluted 1:40000 and an anti-rabbit HRP-conjugated secondary antibody (OriGene, Herford, Germany) diluted 1:1000. The reaction was developed with a chemiluminescence reagent containing luminol (Euroclone, Milan, Italy). Chemiluminescence was developed using LiteAblot^®^ TURBO (Euroclone, Milan, Italy) and exposed on Kodak Biomax film. MMP protein expression was quantified using the ImageJ software, version 1.45s and was reported as percentage with respect to actin.

### 4.6. Cell Line and Stimulation

The THP1 human monocytic cell line (ATCC, TIB-202) was grown in RPMI 1640 medium (Sigma-Aldrich, Saint Louis, MO, USA) supplemented with 10% fetal bovine serum 1% L-glutamine 200 mM, 1% penicillin 10,000 UI/mL, and streptomycin 10 mg/mL. The cell cultures were maintained according to standard procedures in a humidified incubator at 37 °C with 5% CO_2_, and cell passage was performed twice a week. THP1 cells were differentiated into macrophages using 5 ng/mL of phorbol-12-myristate 13-acetate (PMA, Sigma-Aldrich, Saint Louis, MO, USA) for two days. Monocytes (THP1) and macrophages (THP1 + PMA) were stimulated with lipopolysaccharide (LPS; Sigma, Saint Louis, MO, USA) 1 μg/mL for 3 h.

### 4.7. Transient Transfection

The full-length of GAS5 sequence was sub-cloned into pcDNA3.1 plasmid to generate pcDNA3.1-GAS5. The empty pcDNA3.1 plasmid was used as negative control. Shortly before transfection, 5 × 10^5^ cells were seeded in twenty-four-well plates in 500 μL of complete medium. A total of 1 μg of DNA was diluted in 3 μL of TransIT^®^-Jurkat transfection reagent (Mirus Bio, Madison, USA) and, after 10 min of incubation, the complexes were added dropwise onto the cells according to the manufacturer’s protocol. Twenty-four hours after transfection, cells were incubated with fresh RPMI medium and then stimulated as described above. The analysis of GAS5 overexpression was carried out after 24 h using real-time RT-PCR.

### 4.8. MMP2 and MMP9 Quantification

Cell culture supernatant obtained from THP1 cells, transfected with pcDNA3.1-GAS5 and then exposed to LPS, PMA, and PMA + LPS as described above, was analyzed using Human MMP2 and MMP9 ELISA Kit (Sigma-Aldrich, Saint Louis, MO, USA), according to the manufacturer’s instructions.

### 4.9. Statistical Analyses

Statistical analyses were performed using GraphPad Prism version 4.00. Since the distribution of gene expression data in tissues obtained from patients was not parametric, data were normalized by logarithmic transformation and paired t-test was applied. One-way ANOVA or two-way ANOVA with Bonferroni post-test and *t* test were used for the in vitro studies of gene and protein expression; *p*-values <0.05 were considered statistically significant.

## Figures and Tables

**Table 1:** Characteristics and histologic scores of the patients.

**Patients (*n*)**	25
**Age (mean, range)**	12.5, 6.2–18
**Male (%)**	13 (52%)
**CD (%)**	13 (52%)
**UC (%)**	12 (48%)
***Clinical score:***	
**PCDAI ^1^ (mean, range)**	30.6, 7.5–55
**PUCAI ^2^ (mean, range)**	30.1, 10–75
***Histologic findings:***	
**Inflammation score**	Score (% of patients)
	0 (0%)
	1 (4%)
	2 (24%)
	3 (36%)
	4 (28%)
	5 (8%)
**Architectural score**	Score (% of patients)
	0 (0%)
	1 (8%)
	2 (28%)
	3 (52%)
	4 (12%)

^1^ Pediatric Crohn’s Disease Activity Index; ^2^ Pediatric Ulcerative Colitis Activity Index.

## Supplementary Materials

Supplementary materials can be found at https://www.mdpi.com/1422-0067/20/21/5280/s1.

## Abbreviations

lncRNA	Long non-coding RNA
GAS5	Growth arrest–specific transcript 5
MMPs	Matrix metalloproteinases
IBD	Inflammatory bowel disease
CD	Crohn’s disease
UC	Ulcerative colitis
LPS	Lipopolysaccharide
PMA	Phorbol-12-myristate 13-acetate
ECM	Extracellular matrix components
PCDAI	Pediatric Crohn’s Disease Activity Index
PUCAI	Pediatric Ulcerative Colitis Activity Index
