# Supplementary material for: Long Non-Coding RNA GAS5 and Intestinal MMP2 and MMP9 Expression: A Translational Study in Pediatric Patients with IBD
Source: Int J Mol Sci. 2019 Oct 24;20(21):5280. doi: 10.3390/ijms20215280 (PMC6862115; doi:10.3390/ijms20215280)
Supplement: Supplementary file 1 [file ijms-20-05280-s001.pdf]

Table S1. Patients' real-time PCR results

	ΔCt					
Samples	GAS5_NON INFL	GAS5_INFL	MMP2_NON INFL	MMP2_INFL	MMP9_NON INFL	MMP9_INFL
P1	4,620237	6,024159	6,09	3,66	6,02	4,56
P2	4,05109	5,337561	6,359166	6,090326	6,625074	5,583005
P3	4,80853	5,542537	6,28	1,7	6,13	0,76
P4	3,738402	5,134124	7,180901	3,708998	8,762146	4,384336
P5	4,184281	3,964396	4,629323	5,272729	6,780655	4,897943
P6	5,089714	5,110917	5,531791	5,466882	6,215479	5,268142
P7	4,320931	4,424052	5,309913	6,333984	3,718985	6,12
P8	4,215015	3,752263	5,103725	5,335869	8,339451	7,05298
P9	3,408993	4,685316	6,588463	3,055487	6,934928	5,647199
P10	3,692512	3,645735	4,212292	2,962023	3,7453	2,865394
P11	4,409967	4,506217	5,093131	4,436662	5,41565	4,638136
P12	6,3339	5,926348	5,510284	3,819945	4,91098	4,524989
P13	5,458839	5,900442	5,202206	5,441165	5,325829	5,531845
P14	4,671136	4,68776	5,626925	4,574403	6,278955	2,637847
P15	5,873657	5,505826	5,053959	4,710312	4,53785	4,146638
P16	5,064594	4,743629	4,831324	4,517202	4,475673	4,030229
P17	4,493004	4,769635	4,509717	4,328194	5,00594	5,229953
P18	4,389392	5,045495	4,991269	5,463724	6,768676	4,658124
P19	4,744884	5,287046	4,107415	5,282191	5,061945	6,645454
P20	3,262977	3,268515	5,408267	-1,143571	4,917294	-1,078093
P21	2,321003	4,267191	6,187333	2,594765	7,305907	4,975801
P22	4,491241	5,028173	5,502692	5,441613	5,759865	5,124155
P23	3,38788	2,73675	6,217258	5,227576	7,636245	3,046763
P24	4,256607	5,319472	5,398791	5,548339	4,475	2,287226
P25	4,413749	4,594313	4,745641	4,98333	2,8356	3,760706

	2^-ΔCt					
Samples	GAS5_NON INFL	GAS5_INFL	MMP2_NON INFL	MMP2_INFL	MMP9_NON INFL	MMP9_INFL
P1	0,040660253	0,0153655	0,014680043	0,07910979	0,015409886	0,04239389
P2	0,060325426	0,0247306	0,012181486	0,01467673	0,010131039	0,02086162
P3	0,035685208	0,0214551	0,01286861	0,3077861	0,014278616	0,59049633
P4	0,074925364	0,0284757	0,006891812	0,07646811	0,002303198	0,04788322
P5	0,055005474	0,0640616	0,040404982	0,02586726	0,009095351	0,03354071
P6	0,029365907	0,0289375	0,021615485	0,02261021	0,01345719	0,02594963
P7	0,050034569	0,046583	0,025209075	0,01239598	0,075940589	0,01437793
P8	0,053846075	0,0742089	0,029082095	0,02475959	0,003087273	0,00753081
P9	0,094143611	0,0388669	0,010391423	0,1202837	0,008172946	0,01995371
P10	0,077346939	0,0798959	0,053947802	0,12833415	0,074567976	0,13722412
P11	0,047040037	0,0440041	0,029296436	0,04617763	0,023427552	0,04015891
P12	0,012396701	0,0164434	0,021940132	0,07080794	0,033238982	0,04343528
P13	0,02273661	0,0167413	0,027163139	0,02301686	0,024932494	0,02161468
P14	0,039250749	0,0388011	0,020236099	0,04197276	0,012877934	0,16066783
P15	0,017055052	0,022008	0,03010279	0,03819925	0,04304979	0,05645957
P16	0,029881699	0,0373272	0,035125827	0,04367035	0,044945703	0,06120405
P17	0,044409003	0,0366604	0,043897512	0,04978331	0,031121599	0,02664571
P18	0,047715705	0,0302799	0,031439694	0,02265975	0,009171186	0,03960636
P19	0,037294741	0,0256118	0,058015612	0,02569816	0,029936617	0,00998893
P20	0,104170812	0,1037717	0,02354775	2,20927192	0,033093828	2,11124353
P21	0,200128287	0,0519335	0,013722308	0,16553808	0,006319793	0,03177859
P22	0,044463305	0,0306457	0,022055893	0,02300972	0,018454737	0,02867317
P23	0,095531478	0,1500224	0,013440606	0,02668965	0,005026448	0,12101326
P24	0,052315889	0,0250426	0,023702926	0,02136897	0,044966674	0,20486906
P25	0,046916884	0,0413975	0,037275177	0,03161318	0,140087487	0,07377593

	Log2 2-ΔCt					
Samples	GAS5_NON INFL	GAS5_INFL	MMP2_NON INFL	MMP2_INFL	MMP9_NON INFL	MMP9_INFL
P1	-4,620237	-6,024159	-6,09	-3,66	-6,02	-4,56
P2	-4,05109	-5,337561	-6,359166	-6,090326	-6,625074	-5,583005
P3	-4,80853	-5,542537	-6,28	-1,7	-6,13	-0,76
P4	-3,738402	-5,134124	-7,180901	-3,708998	-8,762146	-4,384336
P5	-4,184281	-3,964396	-4,629323	-5,272729	-6,780655	-4,897943
P6	-5,089714	-5,110917	-5,531791	-5,466882	-6,215479	-5,268142
P7	-4,320931	-4,424052	-5,309913	-6,333984	-3,718985	-6,12
P8	-4,215015	-3,752263	-5,103725	-5,335869	-8,339451	-7,05298
P9	-3,408993	-4,685316	-6,588463	-3,055487	-6,934928	-5,647199
P10	-3,692512	-3,645735	-4,212292	-2,962023	-3,7453	-2,865394
P11	-4,409967	-4,506217	-5,093131	-4,436662	-5,41565	-4,638136
P12	-6,3339	-5,926348	-5,510284	-3,819945	-4,91098	-4,524989
P13	-5,458839	-5,900442	-5,202206	-5,441165	-5,325829	-5,531845
P14	-4,671136	-4,68776	-5,626925	-4,574403	-6,278955	-2,637847
P15	-5,873657	-5,505826	-5,053959	-4,710312	-4,53785	-4,146638
P16	-5,064594	-4,743629	-4,831324	-4,517202	-4,475673	-4,030229
P17	-4,493004	-4,769635	-4,509717	-4,328194	-5,00594	-5,229953
P18	-4,389392	-5,045495	-4,991269	-5,463724	-6,768676	-4,658124
P19	-4,744884	-5,287046	-4,107415	-5,282191	-5,061945	-6,645454
P20	-3,262977	-3,268515	-5,408267	1,143571	-4,917294	1,078093
P21	-2,321003	-4,267191	-6,187333	-2,594765	-7,305907	-4,975801
P22	-4,491241	-5,028173	-5,502692	-5,441613	-5,759865	-5,124155
P23	-3,38788	-2,73675	-6,217258	-5,227576	-7,636245	-3,046763
P24	-4,256607	-5,319472	-5,398791	-5,548339	-4,475	-2,287226
P25	-4,413749	-4,594313	-4,745641	-4,98333	-2,8356	-3,760706
